# Cesarean Sections under General Anesthesia at a Tertiary Care Center in Western Nepal: A Descriptive Cross-sectional Study

**DOI:** 10.31729/jnma.4724

**Published:** 2019-12-31

**Authors:** Krishna Murari Adhikari, Gajal Lakhe, Anjali Subedi Adhikari

**Affiliations:** 1 Department of Anesthesiology, Manipal Teaching Hospital, Pokhara, Nepal; 2 Department of Obstetrics and Gynaecology, Manipal Teaching Hospital, Pokhara, Nepal

**Keywords:** *cesarean section*, *elective*, *emergency*, *general anesthesia*

## Abstract

**Introduction:**

General anesthesia is feared to have adverse feto-maternal outcomes compared to neuraxial anesthesia. It is recommended to keep rate of caesarean sections under general anesthesia below 5% and 15% for elective and emergency caesarean sections respectively. This study was conducted to find out the proportion of caesarean sections under general anesthesia at a tertiary care center in western Nepal.

**Methods:**

A descriptive cross-sectional study was conducted among caesarean sections conducted at Manipal Teaching Hospital, Pokhara, Nepal from January 2014 to December 2017. Ethical approval was taken from the Institutional Review Committee with reference number MEMG/IRC/GA/122. All the caesarean sections conducted during this study period were included in the study using whole sampling method. Data for each patient was subsequently entered into an Excel spreadsheet and analyzed using Statistical Package for Social Sciences version 20.

**Results:**

Among 3613 cases, caesarean sections under general anesthesia was observed in 175 (4.84%) in our center over a period of four years at 95% Confidence Interval (4.13-5.55%). The yearly variations ranges from 2.83% to 8.99%. The rate of general anesthesia was found slightly higher in elective 31 (5.82%) as compared to emergency caesarean section 144 (4.67%).

**Conclusions:**

The four year medical records of our institution showed fluctuating trend of caesarian sections under general anesthesia. The rate of general anesthesia for emergency caesarian section was within the recommended rate while it was slightly higher in elective caesarian section.

## INTRODUCTION

There is an increasing trend of Caesarean Sections (CS) in the developing world.^1-3^ CS is commonly performed under Subarachnoid Block (SAB) and it is considered as the gold standard.^4^ However many a times, SAB cannot be performed in all the patients undergoing CS because of lack of time such as in emergency cases, technique failure, contraindications to the technique and patient preferences to General Anesthesia (GA). In such cases ultimately GA is needed to be given.^5,6^

The maternal and neonatal morbidity and mortality rate is high in CS under GA as compared to SAB.^7^ Hence, it is recommended to keep the rate of GA below 5% for elective CS and below 15% for emergency CS.^8^ There are limited publications on CS under GA in Nepal and no such study has been conducted in our centre earlier.

This study aims to find out the proportion of caesarean sections under general anesthesia at a tertiary care center in western Nepal.

## METHODS

This descriptive cross-sectional study was conducted among patients who underwent cesarean sections (CS) at Manipal Teaching Hospital, Pokhara, Nepal from January 2014 to December 2017. Ethical approval was taken from the Institutional Review Committee with reference number MEMG/IRC/GA/122. All the caesarean sections, 3613 conducted during this study period were included in the study using whole sampling method.

Data regarding number of total, elective and emergency CS were obtained from medical record section of the hospital. Medical records of all the CS performed were reviewed to know the number of CS performed under GA. CS who received SAB initially and later supplemented with intravenous and inhalation anesthetic agent with bag and mask ventilation were excluded from the study. The obstetric diagnosis, nature of CS whether elective or emergency, time of the day whether regular or off time of the duty hour were noted.

Data for each patient was subsequently entered into an Excel spread sheet and analyzed using SPSS version 20.

## RESULTS

A total of 3613 cesarean sections were conducted during the study period. Among those cases, caesarean sections (CS) under general anesthesia (GA) were observed in 175 (4.84%) cases in our center over a period of four years at 95% Confidence Interval (4.13-5.55%). Details of total deliveries, CS and CS under GA year wise (Table 1).

**Table 1:** Total number of all deliveries, caesarean sections and cesarean sections under general anesthesia by year.

Year	Total deliveries	CS n (%)	CS under GA n (%)
2014	2466	810 (32.85)	23 (2.83)
2015	2710	634 (23.39)	57 (8.99)
2016	2433	985 (40.48)	47 (4.77)
2017	2211	1184 (53.55)	48 (4.87)
Total	9820	3613 (36.79)	175 (4.84)

In the duration of four years, there were 532 (14.72 %) and 3081 (85.27 %) cases of elective and emergency CS. Similarly, elective CS under GA were observed in 31 (5.82%) cases where as emergency CS under GA were performed in 144 (4.67%) cases (Table 2).

**Table 2:** Elective and emergency caesarean sections under general anesthesia by year.

Year	Elective CS n (%)	Elective CS under GA n (%)	Emergency CS n (%)	Emergency CS under GA n (%)
2014	90 (11.11)	5 (4.44)	720 (88.88)	18 (2.63)
2015	157 (24.76)	12 (7.64)	477 (75.23)	43 (9.84)
2016	116 (11.76)	4 (3.44)	869 (88.22)	43 (4.94)
2017	169 (14.27)	10 (5.91)	1015 (85.72)	40 (3.94)
Total	532 (14.72)	31 (5.82)	3081 (85.27)	144 (4.67)

More number of CS under GA 114 (65.14%) was performed during regular time in comparison to the number during off time 61 (34.9%). A total of 31 (100%) elective CS under GA were done during the regular time, however, there weren't any elective CS under GA during off time. Among 144 emergency CS under GA, 83 (57.6%) cases were performed during regular time whereas 61 (42.36%) cases were performed during off time. Relationship between the rate of CS under GA and time of the duty hour (Table 3).

**Table 3:** Time of the day and its relation to Caesarean section under General anesthesia.

CS under GA (n)	Regular time n (%)	Off time n (%)
All CS (175)	114 (65.1)	61 (34.90)
Elective CS (31)	31 (100)	0
Emergency CS (144)	83 (57.6)	61 (42.36)

## DISCUSSION

The rate of CS under GA during study period of four years was 4.84%. Rate varied from lowest 2.83% in 2014 to highest 8.99 % in 2015, after which the rate decreased to 4.77% in 2016 and again increased to 4.87% in 2017. The rate of CS under GA was 1.87% in a similar study conducted in tertiary care centre of Nepal by Sigdel R et al,^9^ 1.9 % in a study done by Cool E et al^10^ in Belgium and 0.5% to 1.0% in a study done in USA by A. Palanisamy et al.^11^ In our study, there was higher proportion of failed spinal as an indication to GA (62.85% of all CS under GA) in comparison to above studies, which might be the reason for increased rate of CS under GA in our study.

Higher rate of GA for CS (20%-87%) was seen in studies conducted in Singapore, Nigeria and western Africa, Antigua, Bermuda and Turkey compared to our study. Higher proportion of indication as a maternal choice of anesthesia, lack of physician anesthetist trained in neuraxial anesthesia and unfamiliarity with neuraxial technique in the anesthesia provider were the reasons for the high rate of GA for CS.^^5^,^12^-^14^^ The rate of CS under GA has also been seen related to ethnicity, geographical location and region of origin. A study done in Ireland demonstrated high rate of GA for emergency CS in migrants from North Africa, Middle East and Eastern Europe in comparison to the native Irish people.^15^

Slightly higher rate of elective CS were performed under GA compared to emergency CS in our study (5.82% vs. 4.67%), which was opposite to our anticipation that more number of emergency CS might require GA because of lack of time and associated co-morbidities contraindicating SAB. Higher proportion of failed spinal as an indication to GA in elective CS compared to emergency CS (86.66% vs. 60.13%) contributed to this result. The reason for more proportion of failed spinal in elective CS in our centre might be because of resident being involved in the procedure, who performed SAB more in elective cases.

The rate of GA for emergency CS is within that recommended by The Royal College of Anesthetist in its 2012 audit report, while the rate of GA in elective CS is slightly higher than recommended (the recommendation is below 5% for elective CS and below 15% for emergency CS ).^8^ The rate of elective CS under GA varied from 3.44% to 7.64% and the rate of emergency CS under GA ranged from 2.63% to 9.84%. However, in a study conducted by Sari M et al. in Turkey, the use of GA ranged from 10.1% to 30.1% in elective CS and 23.5% to 42.6% in emergency CS.^12^

In our study, out of the total CS performed under GA, 80.57% were emergency cases and 18.05% were elective cases, which is similar to that reported in past studies.^11,12^

More number of CS under GA was performed during regular time in comparison to off time (65.14% vs. 34.9%). While more number of CS were performed in off time, compared to regular time (54.1% vs. 45.9%) in a study done by Planisamy A et al. This difference in two studies is because of different pattern of patient admission in relation to time. In our centre, more number of emergency CS which required GA were admitted on regular time than off time (57.6% vs. 42.36%) which was opposite to their findings.^11^

The reasons of GA for CS were similar to that of other studies, but with varied proportions.^5,10,11,16^ Obstetric co-morbidities and fetal distress with lack of time for neuraxial procedure were the most common indications of GA in study done by Cool E et al. and Palanisamy A.^10,11^ While maternal choice of GA was most common indications in a study done by Kan E et al.^5^ In our study, failed spinal (62.85%) was the most common indication for GA followed by 17.7% cases of Hypertensive disorders of pregnancy and 6.28% cases of ante-partum hemorrhage who were unstable hemodynamically, had abnormal coagulation profile and altered consciousness were started in GA. Six cases of Hypertensive disorder of pregnancy and four cases of APH who were stable hemodynamically converted to GA later because of failed spinal. Though there were 8.57% cases with fetal distress as a reason to CS who received GA, only 1.71% of them were started on GA initially because of severe fetal bradycardia with no time for neuraxial anesthesia. Other cases were converted to GA later because of failed spinal.

This study was designed to observe the frequency of caesarean sections under general anesthesia at a tertiary care center in western Nepal. Other important factors such as feto-maternal outcomes of CS under GA could not be addressed by this study. Since this study was conducted in a single institution; hence the results of the study cannot be generalized.

## CONCLUSIONS

The four year medical records of our institution showed fluctuating trend of caesarian sections under general anaesthesia. The rate of GA for emergency CS was within the recommended rate while it was slightly higher in elective CS.

## Conflict of Interest

**None.**
